# Validity of the Polar V800 heart rate monitor to measure RR intervals at rest

**DOI:** 10.1007/s00421-015-3303-9

**Published:** 2015-12-26

**Authors:** David Giles, Nick Draper, William Neil

**Affiliations:** Department of Life Sciences, College of Life and Natural Sciences, University of Derby, Buxton, 1 Devonshire Road, Buxton, Derbyshire SK17 6RY UK; School of Sport and Physical Education, University of Canterbury, Christchurch, New Zealand

**Keywords:** Heart rate variability, Polar V800, Time domain analysis, Frequency domain analysis, Non-linear analysis

## Abstract

**Purpose:**

To assess the validity of RR intervals and short-term heart rate variability (HRV) data obtained from the Polar V800 heart rate monitor, in comparison to an electrocardiograph (ECG).

**Method:**

Twenty participants completed an active orthostatic test using the V800 and ECG. An improved method for the identification and correction of RR intervals was employed prior to HRV analysis. Agreement of the data was assessed using intra-class correlation coefficients (ICC), Bland–Altman limits of agreement (LoA), and effect size (ES).

**Results:**

A small number of errors were detected between ECG and Polar RR signal, with a combined error rate of 0.086 %. The RR intervals from ECG to V800 were significantly different, but with small ES for both supine corrected and standing corrected data (ES <0.001). The bias (LoA) were 0.06 (−4.33 to 4.45 ms) and 0.59 (−1.70 to 2.87 ms) for supine and standing intervals, respectively. The ICC was >0.999 for both supine and standing corrected intervals. When analysed with the same HRV software no significant differences were observed in any HRV parameters, for either supine or standing; the data displayed small bias and tight LoA, strong ICC (>0.99) and small ES (≤0.029).

**Conclusions:**

The V800 improves over previous Polar models, with narrower LoA, stronger ICC and smaller ES for both the RR intervals and HRV parameters. The findings support the validity of the Polar V800 and its ability to produce RR interval recordings consistent with an ECG. In addition, HRV parameters derived from these recordings are also highly comparable.

## Introduction

Heart rate variability (HRV) is a non-invasive tool, which allows the exploration of cardiovascular autonomic function through the measurement of variations in RR intervals (Thayer et al. 2012). Heart rate variability was first employed in clinical settings (Akselrod et al. 1981; Fouad et al. 1984), before being applied to sport sciences contexts (Seals and Chase 1989). The use and analysis of HRV has become increasingly common, as it is simple, non-invasive and sensitive to physiological and psychological changes (Thayer et al. 2012).

In a clinical setting, reduced HRV has been shown to unfavourably reflect prognoses for cardiovascular disease, diabetic neuropathy, arterial hypertension, acute myocardial infarction and other heart conditions (Spallone et al. 2011; Thayer et al. 2010; Yi et al. 2014). Heart rate variability may also provide an insight into the capacity of an organism to function effectively in complex environmental, physiological and psychological conditions (Thayer et al. 2012). Heart rate variability has been found to be a valuable measure in a variety of sports settings with the measurement of many factors including overtraining, recovery, endurance training, and exercise (Makivić et al. 2013).

Advances in technology have provided athletes, coaches and researchers with an affordable, robust and reliable means of recording RR data in the form of heart rate monitors (HRM) worn on the wrist with wireless chest strap electrodes. Instruments such as Polar’s HRMs (Polar OY, Finland), which are capable of recording RR intervals, are used not only by athletes, but also for HRV analysis in other fields such as sports science and medicine (Gamelin et al. 2006). The development of HRMs has enabled recording of RR data in situations where it was not previously possible with lab based electrocardiograms (ECG), or even ambulatory ECGs (Mateo et al. 2012; Morales et al. 2013). However, these HRM are consumer devices, which are not specifically designed for clinical or research application; as such, the validation their potential to accurately and reliably record RR intervals is essential.

Earlier Polar HRM have been validated, including the S810 (Gamelin et al. 2006, 2008; Nunan et al. 2008, 2009; Porto and Junqueira 2009; Vanderlei et al. 2008; Weippert et al. 2010) and more recently the RS800 (Quintana et al. 2012; Wallén et al. 2012). All studies demonstrated that recordings of RR intervals made by the Polar HRM are in good agreement with ECG systems, with small, but acceptable level of variation when compared to simultaneously recorded 2, 3 or 12 lead ECGs. This also holds true for HRV parameters derived from the RR intervals, as long as both signals are processed with the same software. It has been shown that software differences in signal processing and calculation of HRV indices results in unacceptable variation (Nunan et al. 2008; Radespiel-Tröger et al. 2003; Sandercock et al. 2004; Wallén et al. 2012; Weippert et al. 2010). The present study set out to validate the newly released Polar V800, which supersedes the discontinued S810 and RS800 and has not yet been examined in the literature.

The aim of the study was to assess the validity of the Polar V800 heart rate monitor to accurately measure RR intervals at rest, comparing: (1) resting raw data obtained during supine and standing measurements from the Polar V800 HRM and a 3 lead ECG recording; and, (2) linear and non-linear HRV parameters derived from both the V800 and ECG. The present study also aimed to improve on the methods for the identification and correction of RR intervals employed (Gamelin et al. 2006, 2008), using method representative of typical use in clinical and sports science settings.

## Method

### Participants

Twenty (3 female and 17 male) volunteers (age 28.7 ± 9.9 years; height 1.76 ± 0.09 m; mass 75.9 ± 9.5 kg) agreed to participate in the study. Non-smoking volunteers were selected for participation based on having no known cardiovascular or respiratory diseases or illnesses. No participant was known to be taking medication or have any cardiovascular problems that may have influenced the procedures carried out. Participants completed written informed consent and medical health questionnaires prior to taking part in the study. Approval for the study was granted by the University of Derby’s Ethics Committee [LSREC_1415_16] and conformed to the principles of the declaration of Helsinki.

### Procedure

Participants were asked to abstain from caffeine-containing food and drink prior to the test and to only consume a light meal 2 h prior to testing. Participant’s skin was cleaned (shaved if necessary) and prepared for the attachment of the ECG electrodes. The electrodes were placed in a CM5 configuration [right fifth interspace, manubrium and left fifth interspace (Dash 2002)], ensuring that they did not interfere with the fit of the HRM strap (Polar H7). The electrode belt was dampened and placed following Polar’s guidelines, tightly but comfortably just below the chest muscles. Resting measurements were conducted in two positions, supine and, following an active orthostatic challenge, standing in a quiet laboratory, with a temperature of 20.6 ± 1.0 °C. Recordings lasted for 10 min in the supine position and 7 min in standing position. In order to control for the influences of respiration on HRV (Song and Lehrer 2003) participants matched their breathing frequency to an auditory metronome set at 0.20 Hz (12 breaths min^−1^). No attempt was made to control the participant’s tidal volume (Pöyhönen et al. 2004).

### Data recording

RR interval data were recorded simultaneously using a V800 Polar HRM with a Polar H7 chest strap and a three-lead ECG (MP36, Biopac Systems Ltd.), at a sampling frequency of 1000 Hz for both devices. R-wave peaks from the ECG were detected automatically using a custom peak detection algorithm in Matlab (Mathworks, Cambridge). The raw ECG traces and detected R waves were manually assessed to ensure that they had been correctly detected, missed beats were added in manually. Ectopic beats were noted, but not corrected at that stage of analysis. Data was saved as RR interval data files, with intervals in ms. For the Polar HRM raw unfiltered RR data was exported from the Polar Flow web service as a space delimited.txt file.

### Data analysis—error identification

Both the ECG and HRM raw RR signal start points were manually matched before further analysis. The two signals were compared side-by-side to identify errors greater than 20 ms. Signals were analysed for errors caused by the data recording using the Polar HRM in comparison to the ECG and non-sinus beats. Non-sinus beats were replaced during analysis in both signals with interpolated data from adjacent RR intervals (*N* = 1). Before correction discrepancies between the two signals, were identified and synchronicity maintained with the insertion of a 0 ms interval. Following visual identification, discrepancies were assigned to one of the six types of errors given in Table 1. The errors identified are based on the research of Gamelin et al. (2006, 2008), with the addition of T6 (a and b) error which had not previously been detected (or were not identified) in previous HRM, but were found with the V800 recordings. A T6 error was identified as an RR interval entirely missed by the HRM, two types of T6 errors were labelled: T6-a were not detectable without a simultaneous ECG recording, whilst T6-b were identified by a discrepancy between the time stamp in the first column and the length of the interval in the second column of the.txt file exported from the PolarFlow web service.

**Table 1:** Types of error and methods for their correction

Type of error	Description	Correction for HRM data
T1	A discrepancy greater than 20 ms at a single interval, either positive or negative	Error recorded, but not corrected
T2	A long interval, followed by a short interval. Whilst the two points either side were unaffected (<20 ms)	Two uncorrected R–R intervals averaged
T3	Short interval, followed by a long interval. Whilst the two points either side were unaffected (<20 ms)	Two uncorrected R–R intervals averaged
T4	Missed interval(s) on the HRM, equivalent to two or three ECG RR intervals	RR interval divided by the number of undetected R waves
T5	Extra, short, RR intervals from the HRM, in the space of one on the ECG	RR intervals combined to approach the corresponding ECG value
T6-a	RR interval(s) entirely missed by the HRM, undetectable	Error recorded, but not corrected
T6-b	RR interval(s) entirely missed by the HRM, detectable	Interpolated value from the two adjacent points inserted

### Data analysis—error correction

Once identified, it is necessary to correct errors in the RR time series. Previously, Gamelin et al. (2008) corrected all errors detected (T1–T5), however, this does not represent typical use, as, without the use of a simultaneous ECG recording it is not possible to detect T1 errors; equally, the newly identified T6-a error is undetectable without a simultaneous recording. Several issues with the method employed for the correction and processing of RR intervals were also identified by Nunan et al. (2008), who argued that exporting the Polar and ECG RR intervals to the same spreadsheet and applying the same editing, interpolation, resampling and detrending procedure to both data sets [as were performed by Gamelin et al. (2008) and the present study], rather than using each system’s individual HRV processing capability, is unrealistic and not representative of typical use. Whilst a realistic ‘real-world’ approach to the correction of intervals and calculation of HRV parameters appears logical, the results of previous studies find issue with the use of different HRV processing software, as a large number of studies have shown that differences in HRV analysis software produce parameters with very poor agreement (Nunan et al. 2008; Radespiel-Tröger et al. 2003; Sandercock et al. 2004; Wallén et al. 2012; Weippert et al. 2010); further, polar no longer provide tools for the analysis of HRV through their PolarFlow service and RR intervals must be downloaded and analysed in a separate software package anyway. As such, in order to address some of the previous comments, the present study avoids the correction of unidentifiable errors (T1 and T6-b), whilst still correcting errors identifiable without a simultaneous ECG recording (T2–T5 and T6-b) following the guidelines given in Table 1, before analysing both data sets with freely available software [Kubios HRV, version 2.2 (Tarvainen et al. 2014)]. Following analysis of the Polar RR trace for errors and the replacement of ectopic, erroneous and noisy complexes, the RR interval data was considered normal, and thus described as NN data.

### Data analysis—time and frequency domain and non-linear analysis

For the calculation of HRV parameters an identical 256-s segment of NN intervals was selected from the last 300-s of the ECG and corrected HRM recordings. These selected segments were analysed using Kubios HRV (Version 2.2) for time, frequency domain and non-linear components.

#### Time domain analysis

Time domain analysis concerns the statistical representation of the variation in NN intervals within the sample (Karim et al. 2011). A number of parameters may be calculated: SDNN is the standard deviation of the NN intervals, RMSSD the root mean squared of successive difference of intervals and pNN50 % the number of successive differences of intervals that differ by more than 50 ms, expressed as a % of the total (Karim et al. 2011).

#### Frequency domain analysis

Frequency domain analysis allows for the identification of sympathetic and parasympathetic contributions of HRV. Non-parametric power spectral density (PSD) analysis provides basic information on how power, and therefore the variance, distributes as a function of frequency using a fast Fourier transformation. A fast Fourier transformation allows the analysis of the components of the power spectrum density to be quantified into different frequency bands for further analysis (Achten and Jeukendrup 2003). Three spectral components were calculated, very low frequency (VLF; 0.00–0.04 Hz), low frequency (LF; 0.04–0.15 Hz) and high frequency (HF; 0.15–0.40 Hz). Additionally, normalised LF and HF power were calculated (as a percentage of the sum of LF and HF power) and the ratio LF:HF power.

#### Non-linear analysis

Given the complex control systems of the heart it is reasonable to assume nonlinear mechanisms are involved in the genesis of HRV; non-linear analysis of NN intervals describes the chaotic nature of the signal (Tarvainen et al. 2014). The data were analysed as a Poincare Plot, which is a widely used graphical representation of the correlation between successive NN intervals (Brennan et al. 2001). The analysis comprised of fitting an ellipse oriented according to the line-of-identity and computing the standard deviation of the points perpendicular to and along the line-of-identity, referred as SD1 and SD2, respectively (Brennan et al. 2001). Sample Entropy was also calculated, measuring the complexity of the NN series, low entropy arises from extremely regular time series, higher values reflect more complexity, and highest values are typical for stochastic data sets (Weippert et al. 2014).

### Statistical analysis

Descriptive statistics were first calculated for all variables, all values are reported as mean ± SD. Normal distribution and homogeneity of variance was assessed through visual inspection of the frequency histogram, and with either a Kolmogorov–Smirnov (RR intervals) or Shapiro–Wilk test (HRV parameters) depending on the number of samples. Homoscedasticity was determined through the analysis of the plot of the standardised residuals. Depending on the distribution of data either a Student paired *t* test, or Wilcoxon matched pairs test, was used to determine the differences between the data obtained from the ECG and HRM for both the RR intervals and the calculated HRV parameters. The magnitude of the difference of the RR intervals and the HRV parameters was calculated by determining the effect size (ES) which represents the mean difference over the standard deviation of the difference (Thomas et al. 2010); the difference was considered small when ES ≤0.2, moderate when ES ≤0.5, and great when ES >0.8 (Cohen 2013). Relative reliability was assessed for all variables by calculating the intra-class correlation coefficient (ICC) (Weir 2005), and, as recommended by Atkinson and Nevill (1998), model 3.1 was used. Bland–Altman plots were constructed for supine and standing uncorrected and corrected RR intervals and 95 % limits of agreement (LoA) were calculated for all RR and HRV parameters (Bland and Altman 1986). If heteroscedasticity was present in any HRV data it was log-transformed before the calculation of the LoA. The level for accepting statistical significance of tests was set at *P* < 0.05 for all analysis. All data were analysed using SPSS (Version 22; Chicago, IL, USA).

## Results

The total combined number of RR intervals detected in the supine position was 12247, and in a standing position 11240, with 10 errors detected in each of the positions (Table 2), this corresponds to an error rate of 0.082 and 0.089 %, respectively. A Wilcoxon matched pairs test demonstrated a significant difference between the non-normally distributed supine ECG and corrected Polar RR intervals (*P* < 0.005, ES = 0.000-small), and the uncorrected intervals (*P* < 0.005, ES = 0.001-small). Similarly, a Wilcoxon matched pairs test revealed a significant difference between the non-normally distributed standing corrected ECG and Polar RR intervals (*P* < 0.005, ES = 0.000-small), and the uncorrected intervals (*P* < 0.005, ES = 0.004-small). Effect sizes for the four comparisons were small, <0.004 in all cases.

**Table 2:** Clasification of measurement errors in the Polar V800 HRM signal in supine and standing positions

Type of error	Description of error	Supine	Standing
T1	Single interval of discrepancy	2	1
T2	Long interval and short interval	0	0
T3	Short interval and long interval	0	1
T4	Too few intervals detected	6	1
T5	Too many intervals detected	0	2
T6-a	Interval(s) missed entirely, undetectable	1	5
T6-b	Interval(s) missed entirely, detectable	1	0

**Fig. 1:**
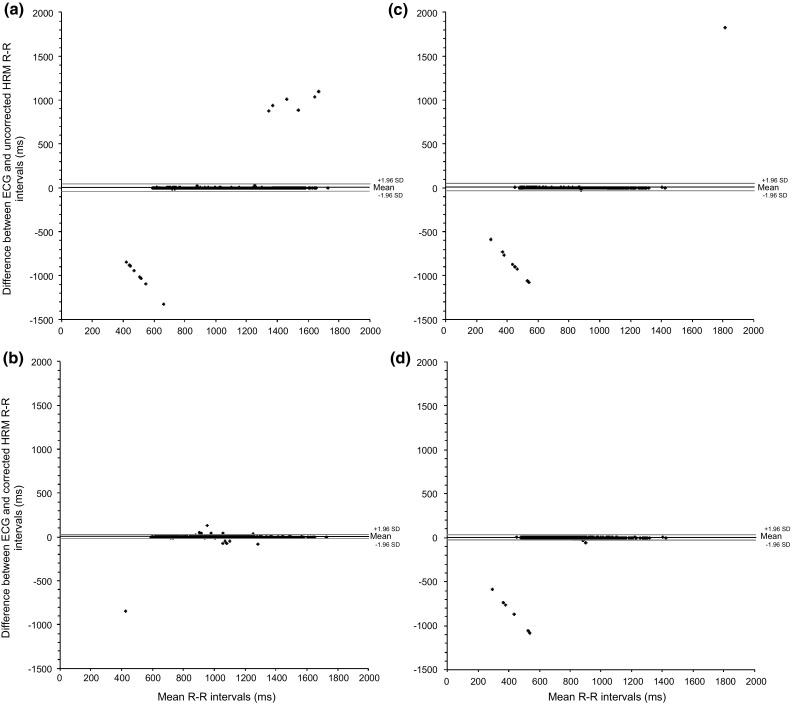
Bland-Altman plots for supine uncorrected (**a**) and corrected (**b**) and standing uncorrected (**c**) and corrected (**d**) ECG and Polar V800 HRM RR interval data

Bland–Altman plots are presented in Fig. 1 for uncorrected and corrected ECG and Polar data. In both the supine and standing positions, the uncorrected and corrected ECG and HRM RR intervals displayed ICCs of 0.982 and 0.975 for uncorrected and 1.00 and 1.00 for the corrected supine and standing data, respectively. Comparisons between the time, frequency and non-linear HRV parameters, derived from the ECG RR and corrected Polar RR intervals with Student paired *t* test did not display any significant differences. Table 3 outlines the bias and limits of agreement (LoA), intra-class correlation coefficients (ICC) and 95 % confidence intervals and effect sizes; effect sizes for the supine and standing HRV data were <0.021 and <0.012, respectively, and were thus classified as small differences.

**Table 3:** Heart rate variability parameters obtained from the ECG and Polar V800 HRM (mean ± SD), bias and limits of agreement (LoA), intra-class correlation coefficients (ICC) and 95 % confidence intervals and effect sizes in supine and standing positions

	ECG (mean ± SD)	Polar (mean ± SD)	Bias (LoA)	ICC (95 % CI)	Effect size (interpretation)
Supine					
SDNN (ms)	61.37 ± 32.00	61.36 ± 32.03	0.01 (–0.22 to 0.24)	1.00 (1.00–1.00)	0.000 (small)
RMSSD (ms)	55.92 ± 37.76	55.92 ± 37.81	0.00 (–0.32 to 0.32)	1.00 (1.00–1.00)	0.000 (small)
PNN50 (%)	29.05 ± 23.05	29.30 ± 23.04	–0.25 (–1.20 to 0.70)	1.00 (1.00–1.00)	0.011 (small)
VLF power (ms^2^)	819.17 ± 777.33	820.05 ± 779.10	–0.88 (–7.52 to 5.76)	1.00 (1.00–1.00)	0.001 (small)
LF power (ms^2^)	1050.87 ± 994.29	1051.82 ± 994.94	–0.95 (–6.25 to 4.36)	1.00 (1.00–1.00)	0.001 (small)
HF power (ms^2^)	182.97 ± 2212.28	1826.52 ± 2216.23	0.45 (–27.95 to 28.84)	1.00 (1.00–1.00)	0.000 (small)
nuLF power	40.97 ± 15.93	41.05 ± 16.07	–0.08 (–0.72 to 0.56)	1.00 (1.00–1.00)	0.005 (small)
nuHF power	58.88 ± 15.88	58.81 ± 16.02	0.08 (–0.57 to 0.72)	1.00 (1.00–1.00)	0.005 (small)
LF:HF ratio	1.00 ± 1.43	1.05 ± 1.63	–0.04 (–0.43 to 0.35)	0.99 (0.98–1.00)	0.029 (small)
SD1	44.96 ± 33.54	44.95 ± 33.57	0.01 (–0.21 to 0.23)	1.00 (1.00–1.00)	0.000 (small)
SD2	83.98 ± 46.25	83.96 ± 46.27	0.02 (–0.20 to 0.24)	1.00 (1.00–1.00)	0.000 (small)
Sample entropy	1.46 ± 0.31	1.46 ± 0.31	–0.01 (–0.11 to 0.09)	0.99 (0.97–1.00)	0.021 (small)
Standing					
SDNN (ms)	52.03 ± 16.67	52.00 ± 16.67	0.02 (–0.17 to 0.22)	1.00 (1.00–1.00)	0.001 (small)
RMSSD (ms)	30.09 ± 18.24	30.06 ± 18.14	0.03 (–0.28 to 0.34)	1.00 (1.00–1.00)	0.002 (small)
PNN50 (%)	7.23 ± 7.84	7.26 ± 7.99	–0.04 (–1.42 to 1.34)	1.00 (0.99–1.00)	0.005 (small)
VLF power (ms^2^)	923.78 ± 766.72	923.99 ± 765.73	–0.20 (–5.58 to 5.17)	1.00 (1.00–1.00)	0.000 (small)
LF power (ms^2^)	1371.91 ± 1132.93	1371.33 ± 1133.29	0.58 (–6.67 to 7.83)	1.00 (1.00–1.00)	0.001 (small)
HF power (ms^2^)	652.26 ± 753.96	647.93 ± 742.37	4.33 (–25.08 to 33.74)	1.00 (1.00–1.00)	0.006 (small)
nuLF power	70.14 ± 13.21	70.19 ± 13.13	–0.05 (–0.83 to 0.74)	1.00 (1.00–1.00)	0.004 (small)
nuHF power	29.75 ± 13.14	29.70 ± 13.06	0.05 (–0.73 to 0.83)	1.00 (1.00–1.00)	0.004 (small)
LF:HF ratio	3.22 ± 2.39	3.23 ± 2.44	–0.01 (–0.35 to 0.33)	1.00 (0.99–1.00)	0.004 (small)
SD1	21.31 ± 12.92	21.29 ± 12.85	0.02 (–0.20 to 0.24)	1.00 (1.00–1.00)	0.002 (small)
SD2	70.16 ± 20.85	70.13 ± 20.88	0.03 (–0.23 to 0.28)	1.00 (1.00–1.00)	0.001 (small)
Sample entropy	1.05 ± 0.28	1.05 ± 0.28	0.00 (–0.05 to 0.05)	1.00 (0.99–1.00)	0.012 (small)

## Discussion

In this present study raw RR intervals and HRV parameters derived from a Polar V800 HRM and a three-lead ECG were compared. The results suggest that the Polar V800 can produce RR interval recordings consistent with an ECG and that the HRV parameters derived from these recordings are comparable, in healthy subjects during a paced active orthostatic test.

### Validity of the detected RR intervals

A significant difference existed between both the corrected V800 and ECG RR intervals and the uncorrected V800 and ECG RR intervals; the significant differences are likely due to the very large sample size of 12247 intervals in the supine position, and 11240 intervals in the standing position, as the effect sizes were small in all cases (uncorrected 0.001 and 0.004, respectively; corrected <0.001 for both). The bias (95 % CI and LoA) of the V800 RR intervals was 0.23 (±66.19; −65.96 to 66.43 ms) and 0.06 (±4.39; −4.33 to 4.45 ms) for uncorrected and corrected supine data, respectively; similarly, the standing intervals were 0.50 (±57.00; −56.50 to 57.50 ms) and 0.59 (±2.28; −1.70 to 2.87 ms) for uncorrected and corrected standing data, respectively. Further, the correction of the Polar HRM RR intervals may be considered highly successful, with a decrease in bias and smaller LoA and an improvement in the ICC from 0.982 (95 % CI 0.981–0.983) to 1.00 (95 % CI 1.00–1.00) and 0.976 (95 % CI 0.975–0.976) to 1.00 (95 % CI 1.00–1.00) for supine and standing intervals, respectively.

The most commonly detected errors (Table 2) were T4 (too few intervals detected) and T6-a (interval missed entirely, undetectable). It is not possible to determine the source of the above errors, but, as thought by Gamelin et al. (2006), it is probable that the T4 errors occurred because of a loss, or decrease in, contact between the skin and the electrode and a resulting reduction in the amplitude of the R wave. Errors in the T6 category (a and b) had not previously been detected when using Polar HRM, it is possible that they result because of software error due to a time asynchronicity in the HRM and/or because of a loss, or decrease in, contact between the skin and the electrode. The T6a error is undetectable without a simultaneous ECG recording, and as such was not corrected; conversely, the T6b error is visible in the.txt RR interval file exported from the PolarFlow website as a discrepancy between the time stamp in the first column and the length of the interval in the second column and, as such, is correctable. With the exception of T1 and T6a error, all other types of error may (and should) be recognised and corrected without the use of a simultaneous ECG recording; the correction of intervals is typical of normal use in a research setting, although further research is required to validate the most appropriate technique for the correction of RR time series. The uncorrected T1 and T6-a errors are visible on the corrected Bland–Altman plots (Fig. 1) as outliers; it is worth noting that in real-world usage, without a reference ECG signal, these would not appear as large discrepancies in the RR time series.

### An improvement in RR detection over previous devices

The combined supine and standing error rate of 0.086 % RR interval detection of the Polar V800 was an improvement on previous Polar HRMs: Gamelin et al. (2006) reported an error rate of 0.40 % with the S810 in adults, Vanderlei et al. (2008) a rate of 6.93 % with the S810i, Gamelin et al. (2008) an error rate of 0.28 % with the S810 in children and Kingsley et al. (2004) an error rate of 0.32 % with the 810 s. The bias of the corrected intervals (0.06 and 0.59 ms for supine and standing, respectively), was small and was also an improvement on those recorded previously: Gamelin et al. (2006, 2008) who recorded a bias and LoA for the Polar S810 as 0.9 ± 12 ms in adults and 0.8 ± 10.4 ms in children; Kingsley et al. (2004) with limits of agreement of −5.92 to 5.89 ms for the Polar 810 s at rest; Nunan et al. (2009) bias of 2.5 ms (±61.8 ms) in the Polar S810; and Porto and Junqueira (2009) with a mean supine difference of 1.85 ms (−6.3 to 2.67 ms) and standing mean difference of −0.7 ms (−3.89 to 2.50 ms).

The small bias, tight limits of agreement, small effect size and large ICC of the ECG and Polar RR data suggests that the Polar V800 HRM is a valid tool for the detection of RR intervals at rest in both supine and standing positions. Any differences that are present are likely due to a combination of the use of the elasticated chest strap, which is not secured in position; differences in the means of the transmission of the data, with Bluetooth signal in the V800, and wired electrodes in the ECG; and differences in the R-wave peak detection algorithms used.

### Validity of derived HRV parameters

The Polar V800 and ECG displayed excellent agreement between time, frequency and non-linear HRV parameters, similar to levels of agreement found in previous research with the Polar S810 (Gamelin et al. 2006, 2008; Nunan et al. 2009) and S810i (Vanderlei et al. 2008). In contrast, poor agreement has previously been found with the Polar Advantage (Radespiel-Tröger et al. 2003), S810 (Kingsley et al. 2004; Nunan et al. 2008), S810i and Suunto t6 (Weippert et al. 2010) and the RS800 (Wallén et al. 2012). It is apparent that the difference between the studies that have found good agreement between devices, and those that did not, is most likely the result of software: HRV parameters derived from differing software packages are simply incomparable. As such comparisons in the present study will be limited to discussing levels of agreement between the V800 and ECG-derived HRV parameters, and general similarities and trends found in previous studies that did not find poor agreement because of software difference (Gamelin et al. 2006, 2008; Nunan et al. 2009; Vanderlei et al. 2008).

When time domain HRV parameters (SDNN, RMSSD and PNN50) derived from the ECG and corrected Polar RR intervals were compared, excellent agreement were found with small bias, ICC in all cases equal to 1.00 regardless of body position and small effect size (<0.029). There were no significant differences in any parameters, including RMSSD, which had previously been found to be significant for the S810 by Gamelin et al. (2006). As RMSSD reflects short-term variability within the data, it is likely that the lower error rate in the present study (0.086 vs. 0.40 %) resulted in fewer differences in short-term variability, which in turn resulted in greater ICC and smaller effect size. Frequency domain components of VLF, LF, HF power, normalised power and LF:HF also displayed excellent agreement in supine and standings positions. As with both the Polar S810 and S810i (Gamelin et al. 2006, 2008; Vanderlei et al. 2008) there were no significant differences in frequency domain parameters, ICCs of frequency parameters were >0.99 and effect sizes <0.029 in all cases. The non-linear measures of SD1, SD2 and sample entropy, as with the time and frequency measures, displayed good agreement for both supine and standing, with ICC of at least 0.99 for all and effect sizes <0.029. No significant differences in any non-linear components were found, in contrast to the significant difference found in SD1 in Gamelin et al. (2006) and SD2 in Gamelin et al. (2008).

The strong ICCs, alongside the small magnitude of bias and LoA and small effect sizes confirm the validity of HRV parameters derived from the corrected V800 HRM RR intervals for HRV analysis. The HRV parameters bias, ICC and ES appear to support an improvement in the V800 over previous HRM models; as the RR intervals, which the HRV parameters were derived from, displayed very good agreement, and the two signals were analysed with the same software the very small differences found were to be expected. Any differences that did exist are likely because of the very small difference between the ECG RR and Polar V800 intervals. Researchers should be cautious about making comparisons between HRV parameters derived from different software packages, particularly when software packages such as Kubios HRV are freely available that support data exported directly from a large number of ECGs, as well as RR interval data (Tarvainen et al. 2014).

## Conclusion

In conclusion, the strong ICC, small bias and tight LoA and small ES found between the ECG and Polar RR data suggest that the Polar V800 HRM is a valid tool for the detection of RR intervals at rest. The Polar V800 also appears to improve on previous HRM models with regard to measurement against ECG. The correction of Polar V800 RR intervals is recommended in order to decrease both the bias and LoA, as it is not only simply applied, but also possible without the simultaneous recording from an ECG. Furthermore, the small bias, narrow LoA, strong ICC (≥0.99), and small ES (≤0.029) also support the use of HRV parameters derived from the corrected Polar V800 signal.

## Abbreviation

ECG: Electrocardiograph(y)
ES: Effect size
HF: High frequency
HRM: Heart rate monitor
HRV: Heart rate variability
ICC: Intra-class correlation coefficients
LF: Low frequency
LFHF: Low frequency to high frequency ratio
LoA: Limits of agreement
NN: Normal to normal
nuHF: Normalised high frequency power
nuLF: Normalised low frequency power
T(1-6b): Error type 1 to 6b
VLF: Very low frequency
